# Etiology and outcomes of current posterior chamber phakic intraocular lens extraction

**DOI:** 10.1038/s41598-020-78661-z

**Published:** 2020-12-10

**Authors:** Hideki Hayakawa, Kazutaka Kamiya, Wakako Ando, Masahide Takahashi, Nobuyuki Shoji

**Affiliations:** 1 Department of Ophthalmology, School of Medicine, Kitasato University, Kanagawa, Japan; 2 Visual Physiology, School of Allied Health Sciences, Kitasato University, 1-15-1 Kitasato, Minami, Sagamihara, Kanagawa 252-0373 Japan

**Keywords:** Diseases, Medical research

## Abstract

This study was aimed to review the etiology and the outcomes of current posterior chamber phakic intraocular lens (Visian ICL, STAAR Surgical) extraction. This review comprised 770 eyes of 403 consecutive patients undergoing ICL extraction. We evaluated prevalence, etiology, uncorrected distance visual acuity (UDVA), corrected distance visual acuity (CDVA), predictability, and patient satisfaction. ICL extraction was required in 8 of 770 (1.0%) eyes. The most common reason was the progression of the pre-existing cataract formation in 5 eyes (63%), followed by residual refractive errors in 3 eyes (38%). Of the 7 eyes targeted for emmetropia, 7 (100%) and 6 (86%) achieved UDVAs of 20/40 and 20/20 or better, respectively. Three eyes (38%) showed no change in CDVA, 3 eyes (38%) gained 1 line, 2 eyes (25%) gained 3 or more lines. 88% and 100% were within ± 0.5 and 1.0 diopter (D), respectively, of the targeted correction. Patient satisfaction improved significantly, from 3.0 ± 1.4 preoperatively, to 8.0 ± 2.4 postoperatively. No vision-threatening complications occurred. ICL extraction was required in approximately 1% of ICL-implanted eyes. Visual and refractive outcomes were good, and patient satisfaction was overall high, even in ICL-extracted eyes.

## Introduction

The EVO Visian Implantable Collamer Lens (ICL; STAAR Surgical, Monrovia, CA, USA), a posterior chamber phakic intraocular lens, has become broadly acknowledged as a safe and effective treatment of moderate to high ametropia, over a long period of time^1–4^. Although the major adverse events of this surgery include pupillary block^5,6^ and cataract formation^7–11^, these complications are considerably decreased by the introduction of the current ICL models with a central port (KS-AP, V4c and V5 models)^12,13^. Nevertheless, some patients underwent ICL extraction in daily practice, even using the current ICL models, especially when a long period of time have passed. The etiology and the outcomes of ICL extraction have not so far been fully elucidated, especially when using the currently available ICL model with a central port in a clinical setting. It may give us essential insights on understanding the recent prognosis of ICL extraction itself in depth. The goal of the current study is to retrospectively review the cause and the prognosis of overall ICL extraction followed by implantation of another ICL or intraocular lens (IOL), in a large cohort of patients undergoing current ICL implantation.

## Results

### Demographics

Table 1 summarizes the preoperative and postoperative demographics of the study population. ICL extraction was required in a total of 8 out of 770 (1.0%) eyes. The mean patient age was 41.0 ± 10.7 years. In 7 (88%) and 1 (13%) of the 8 eyes, we implanted a model V4c ICL and a model V5 ICL, respectively. The follow-up duration was 4.1 ± 2.3 years. Intervals from ICL implantation to extraction were 2.2 ± 1.9 years.

**Table 1:** Preoperative and postoperative demographics of the study population requiring implantable collamer lens (ICL) extraction.

Case	Age	Gender	Model	Etiology	Secondary Intervention	Preoperative					Preoperative				
						logMAR UDVA	logMAR CDVA	Sphere	Cylinder	Ax	logMAR UDVA	logMAR CDVA	Sphere	Cylinder	Ax
1	59	M	V4c	Progression of pre-existing cataract	PEA + IOL implantation	0.22	0.00	− 1.25	0		− 0.30	− 0.30	0.25	− 0.5	10
2	39	F	V4c	Progression of pre-existing cataract	PEA + IOL implantation	0.52	0.52	0	0		0.82	− 0.18	− 2	0	
3	33	M	V4c	Progression of pre-existing cataract	ICL implantation	− 0.08	− 0.18	0.75	− 1.5	180	− 0.18	− 0.30	0.5	− 1	180
4	53	F	V4c	Progression of pre-existing cataract	PEA + IOL implantation	− 0.18	− 0.18	0.25	− 0.5	180	− 0.18	− 0.30	− 0.25	0	
5	46	M	V4c	Progression of pre-existing cataract	PEA + IOL implantation	0.40	− 0.18	− 1.75	− 0.5	10	0.15	− 0.18	− 0.25	− 1.5	165
6	35	M	V4c	Undercorrection	ICL implantation	0.10	− 0.18	− 0.75	0		− 0.30	− 0.30	0	0	
7	35	M	V4c	Undercorrection	ICL implantation	0.15	− 0.30	− 1.25	0		− 0.08	− 0.30	− 0.25	− 0.5	15
8	28	M	V5	Overcorrection	ICL implantation	− 0.18	− 0.30	0.75	− 0.5	90	− 0.18	− 0.30	0	− 0.5	20

*M* male, *F* female, *PEA* phacoemulsification, *IOL* intraocular lens, *ICL* implantable collamer lens, *logMAR* logarithm of minimal angle of resolution, *UDVA* uncorrected distance visual acuity, *CDVA* corrected distance visual acuity.

### Etiology

**Figure 1:**
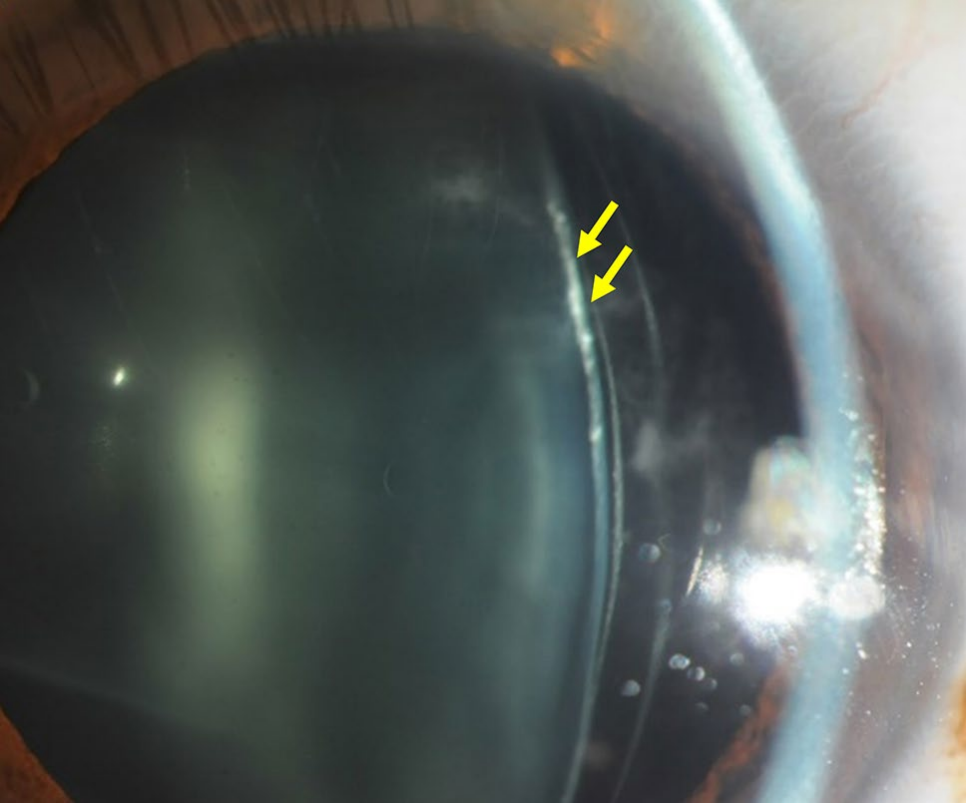
A representative photograph of anterior subcapsular cataract formation in the peripheral area after implantable collamer lens (ICL) implantation.

The most common reason for ICL extraction was the progression of the pre-existing cataract formation in 5 eyes (63%)(Group 1; Cases 1 to 5), followed by residual refractive errors in 3 eyes (38%)(undercorrection in 2 eyes, and overcorrection in 1 eye)(Group 2; Cases 6 to 8). In Group 1, 2 eyes developed nuclear cataract, whereas 3 eyes developed anterior subcapsular cataract, in the peripheral area (1 eye) and in the paracentral area (2 eyes) (Fig. 1). In Group 2, all 3 eyes had refractive errors immediately after initial surgery, due to inaccurate ICL power calculation. In Group 1, cataract surgery with IOL implantation was performed in 4 eyes, and ICL size change (1 size up) due to the mechanical contact in the periphery between the ICL and the crystalline lens was performed in 1 eye. In Group 2, ICL exchange to change the ICL power was performed in all eyes having residual refractive errors.

### Visual and refractive outcomes

**Figure 2:**
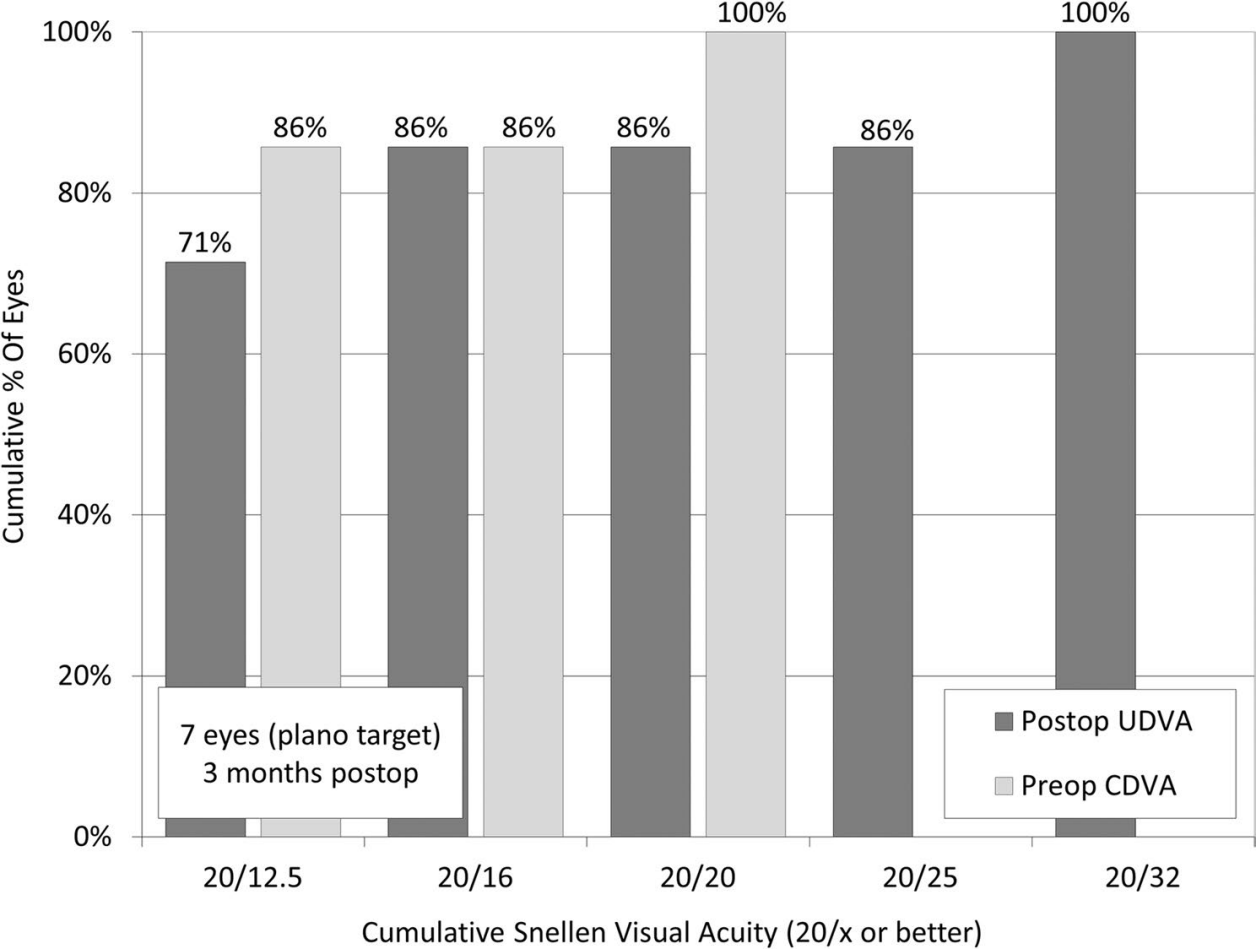
Cumulative percentages of eyes attaining specified cumulative levels of uncorrected distance visual acuity (UDVA) after implantable collamer lens (ICL) extraction.

**Figure 3:**
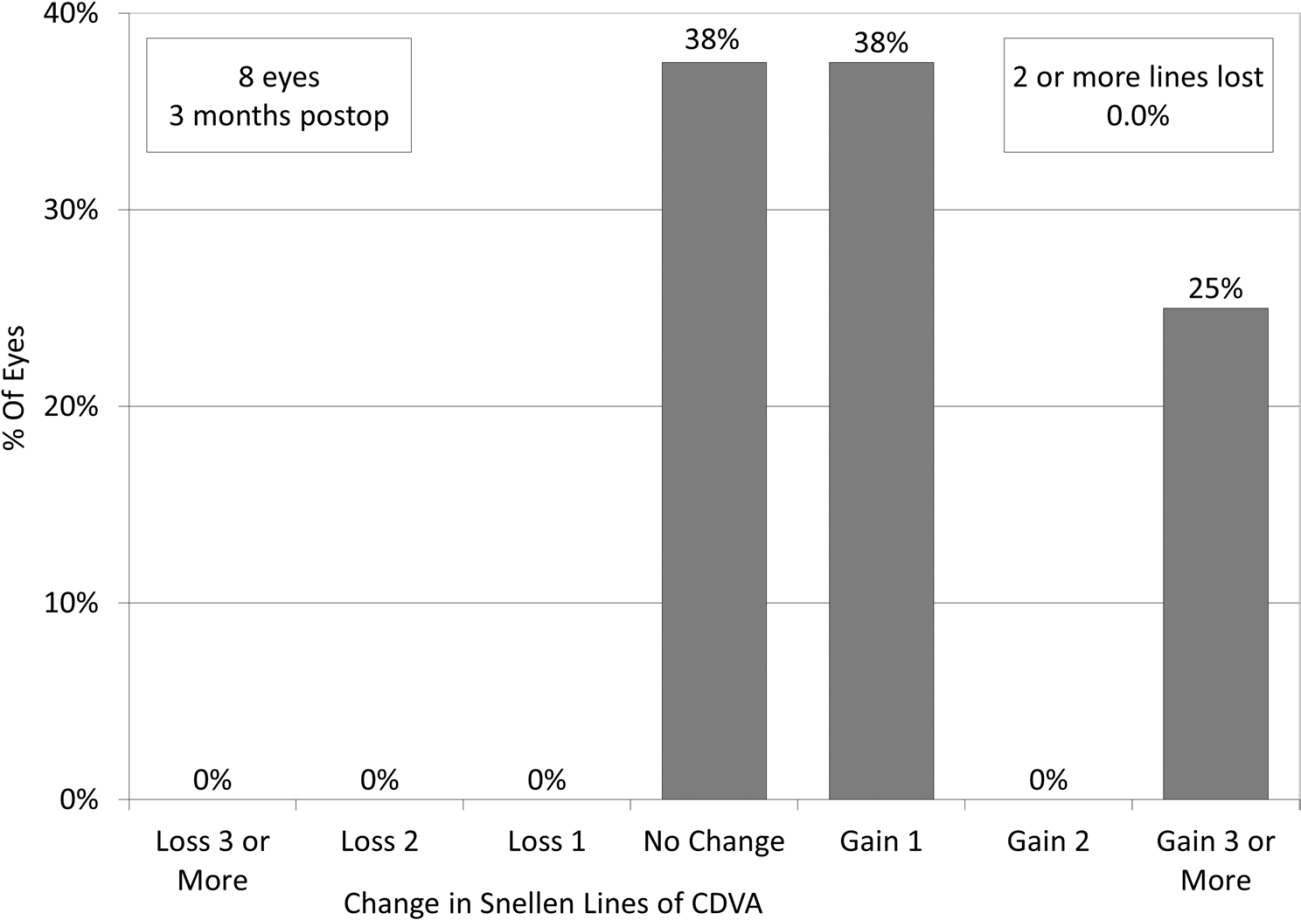
Changes in corrected distance visual acuity (CDVA) after implantable collamer lens (ICL) extraction.

Logarithmic minimum angle of resolution (logMAR) uncorrected distance visual acuity (UDVA) improved, but not significantly, from 0.12 ± 0.26 preoperatively, to − 0.03 ± 0.37 postoperatively (Wilcoxon signed-rank test, p = 0.173). Of the 7 eyes targeted for emmetropia, 7 (100%) and 6 (86%) achieved UDVAs of 20/40 and 20/20 or better, respectively (Fig. 2). LogMAR corrected distance visual acuity (CDVA) improved significantly, from − 0.10 ± 0.27 preoperatively, to − 0.27 ± 0.06 postoperatively (p = 0.043). Three eyes (38%) showed no change in CDVA, 3 eyes (38%) gained 1 line, 2 eyes (25%) gained 3 or more lines, and no eyes lost any lines (Fig. 3). In Group 1, 3 eyes that gained 1 line or more in CDVA underwent cataract surgery and IOL implantation, due to visually significant cataract formation. In Group 2, 3 eyes that gained one line or less underwent ICL exchange, due to incorrect ICL power.

**Figure 4:**
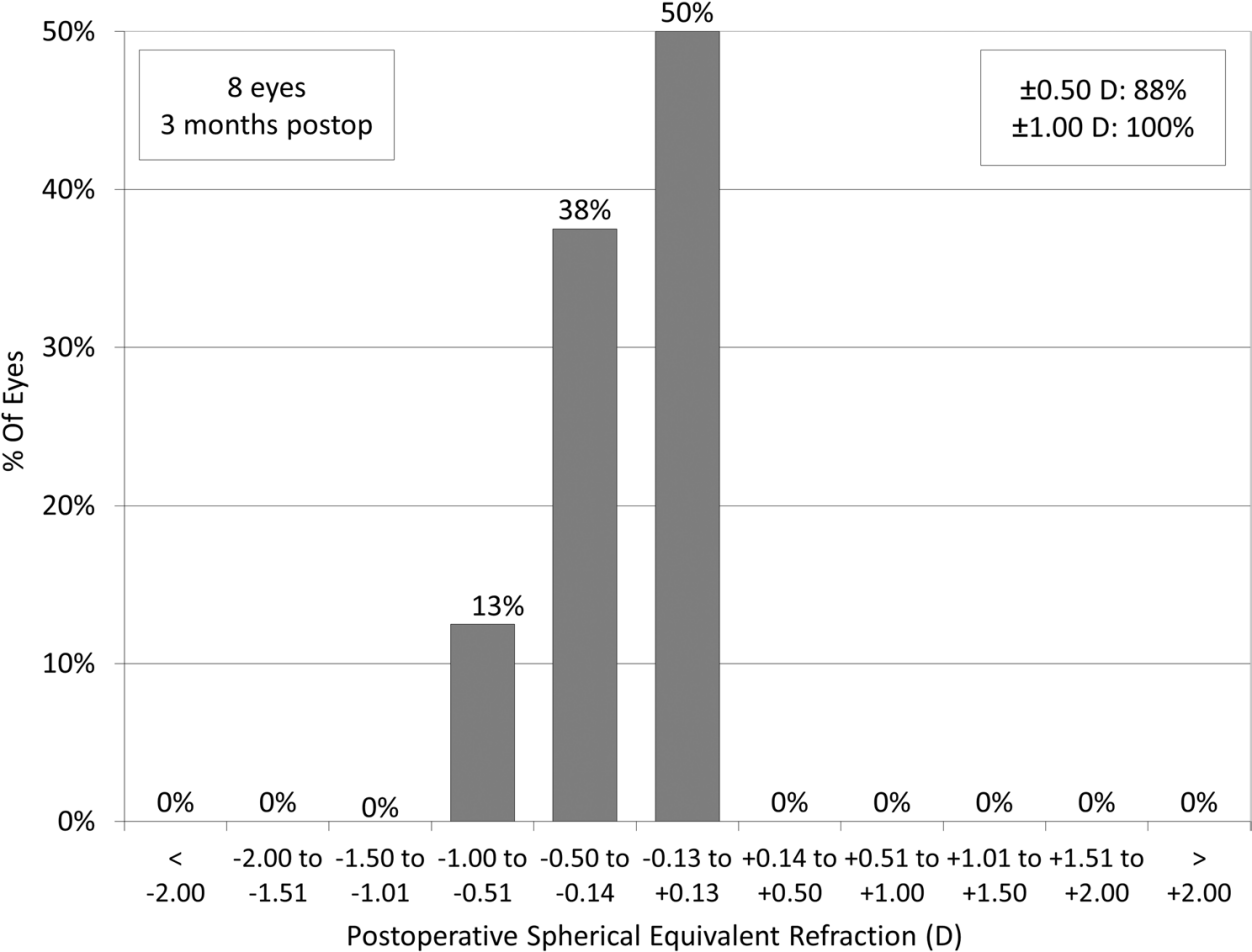
Percentages of eyes within different diopter ranges of the attempted spherical equivalent correction after implantable collamer lens (ICL) extraction.

The targeted refraction was set at emmetropia in 7 eyes for far vision, and at − 2 diopters (D) in 1 eye for near vision. The manifest spherical equivalent did not significantly change, from − 0.44 ± 0.83 D preoperatively, to − 0.50 ± 0.69 D postoperatively (p = 0.917). 88% and 100% of the eyes were within ± 0.5 and 1.0 D, respectively, of the targeted correction (Fig. 4). 80% and 100% of the eyes were within ± 0.5 D of the targeted correction, in Groups 1 and 2, respectively.

### Patient satisfaction

The overall satisfaction score with visual outcomes improved significantly, from 3.0 ± 1.4 preoperatively, to 8.0 ± 2.3 postoperatively (p = 0.043).

### Adverse events

The endothelial cell density (ECD) was not significantly changed, from 2599 ± 372 cells/mm^2^ preoperatively, to 2582 ± 419 cells/mm^2^ postoperatively (p = 0.500). All surgeries were uneventful, and no significant complications, such as cataract progression, significant intraocular pressure rise (> 22 mmHg), pigment dispersion glaucoma, pupillary block, uveitis, posterior capsular opacity, cystoids macular edema, or retinal detachment, were seen at any time in this series.

## Discussion

In the current study, our results demonstrated that ICL extraction was rare, but did exist in approximately 1% after implantation of the current ICL model with a central port, and that the most common reason for ICL extraction was the progression of the pre-existing cataract formation, followed by the residual refractive errors. As far as we can ascertain, this is the first study on the detailed prevalence and etiology of ICL extraction, in a large cohort of patients undergoing implantation of the current ICL model. Our results also demonstrated that visual and refractive outcomes of ICL extraction and subsequent ICL or IOL implantation were good, without significant complications, and thus yielded high patient satisfaction in this series. We assume that the rate of ICL explantation would be rare, but would be required in some eyes having current ICL implantation. We should be aware that a pre-existing cataract might progress over time, and thus such patients should be excluded from candidates for ICL surgery.

Previous studies on ICL extraction and subsequent cataract surgery in eyes having ICL implantation are summarized in Table 2^7–11^. Sanders et al. reported that the incidence of anterior subcapsular opacities with the V3 model (12.6%) was significantly higher than the V4 ICL model (2.9%), and that the need for ICL replacement with the V3 (5.7%) was significantly higher than the V4 ICL (1.1%)^7^. Bleckmann et al. described that 4 of 28 hyperopic eyes (14.3%) developed subcapsular central opacification whereas 5 of 99 myopic patients (5.1%) developed opacifications in ICL-implanted eyes^8^. Morales et al. stated that logMAR UDVA improved from 0.83 ± 0.34 preoperatively to 0.40 ± 0.27 postoperatively, and that logMAR CDVA changed from 0.28 ± 0.19 preoperatively to 0.27 ± 0.21 postoperatively^9^. We previously showed that logMAR CDVA was significantly improved from 0.19 ± 0.30 preoperatively to − 0.06 ± 0.07 postoperatively, and that the percentage of endothelial cell loss was 8.9 ± 11.0%^10^. Gimbel et al. demonstrated that 46 (2.8%) of 1653 eyes underwent ICL removal with cataract extraction and IOL placement as a result of anterior subcapsular cataract^11^. However, all previous studies have merely focused on the incidence and the outcomes of conventional ICL extraction of earlier models without a central port (V2, V3, and V4 models). Fujisawa et al. showed that ICL with a central port was effective for reducing the incidence of cataract formation, presumably by improving the circulation of the aqueous humour to the anterior surface of the crystalline lens^14^. Packer et al. showed that the incidence of asymptomatic anterior subcapsular cataract opacities after current ICL implantation was 0.49% in a total of 617 eyes with a weighted average follow-up of 13 months in 11 previous studies^15^. Indeed, we included not only the 4 eyes (50%) requiring ICL extraction and subsequent cataract surgery, but also the 4 eyes (50%) requiring ICL extraction and subsequent ICL re-implantation, in the current study. In Group 1, the mean age was 46.0 ± 10.4 years, which was considerably higher than that of eyes undergoing ICL implantation. These findings indicate that a higher patient age is one of the possible risk factors for developing cataract, even in current ICL-implanted eyes. In Group 2, all eyes had residual refractive errors immediately after surgery, due to incorrect ICL power calculation, but not due to myopic regression after initial surgery. These findings indicate that ICL exchange showed good predictability outcomes, since all eyes were within ± 0.5 D in Group 2. ICL power is theoretically calculated on the basis of manifest refraction, and thus it is clinically important to accurately determine manifest refraction, especially in higher levels of myopia. Both the incidence and the prognosis are considerably different among these current and previous studies, but the results should be interpreted with some caution (conventional ICL implantation without a central port vs. current ICL implantation with central port, subsequent IOL implantation vs. subsequent IOL or ICL implantation).

**Table 2:** Previous studies on the visual and refractive outcomes of implantable collamer lens (ICL) extraction.

Author	Year	Eyes	Model	Prevalence	F/U (years)	UDVA	CDVA	Within 0.5 D (%)	Within 1.0 D (%)
Sanders et al.^7^	2002	48	V4	1.3%	1.4 ± 0.6	N.A	N.A	N.A	N.A
Bleckmann et al.^8^	2005	14	V4	7.1%	2.6 ± 0.9	N.A	N.A	66%	100%
Morales et al.^9^	2006	12	V4	3.8%	> 0.5	0.40 ± 0.27	0.27 ± 0.21	N.A	71.4%
Kamiya et al.^10^	2010	10	V2,3,4	N.A	7.8 ± 2.8	0.26 ± 0.30	− 0.06 ± 0.07	80%	90%
Gimbel et al.^11^	2018	46	V4	2.7%	2 to 14	N.A	N.A	N.A	N.A
Current	2020	8	V4c, 5	1.0%	2.2 ± 1.9	− 0.03 ± 0.37	− 0.27 ± 0.06	88%	100%

*F/U* follow up, *UDVA* uncorrected distance visual acuity, *CDVA* corrected distance visual acuity, *D* diopter, *N.A.* not available.

There are at least two limitations to this study; one is that this study was performed in a retrospective fashion. Another limitation is that the sample size was kept rather limited, since we experienced only 8 (1.0%) eyes requiring ICL extraction between May 2016 and December 2019. Although we accept that a further study with a large sample size is necessary to confirm our findings, we believe that this information will be helpful for understanding the actual clinical characteristics of ICL extraction, not only for surgeons but also for refractive candidates.

In summary, our findings may support the view that ICL extraction was rare, but did exist in approximately 1% in ICL-implanted eyes, that the most common reason for ICL extraction was the progression of the pre-existing cataract formation, and that the outcomes of ICL extraction and subsequent IOL or ICL implantation were good in terms of safety, efficacy, and predictability, and thus yielded high patient satisfaction.

## Methods

### Study population

We registered the study protocol with the University Hospital Medical Information Network Clinical Trial Registry (000040323). This retrospective clinical chart review comprised a total of 770 eyes of 403 consecutive patients (mean age ± standard deviation, 41.0 ± 10.7 years) who underwent ICL implantation (ICL models; V4c and V5) between May 2016 and December 2019 at Kitasato University Hospital for the correction of moderate to high myopia and myopic astigmatism. Both V4c and V5 models had a central port, but the optics size of V5 was slightly (by approximately 0.1 to 0.3 mm) larger than that of V4c according to the ICL power. The inclusion criteria for ICL surgery at our institution were as follows: unsatisfactory correction with spectacles or contact lenses, 20 ≤ age ≤ 59 years, stable refraction, − 3.00 to − 14.0 D of myopia with astigmatism of 3 D or less, anterior chamber depth ≥ 2.8 mm, ECD ≥ 1800 cells/mm^2^, no history of ocular surgery, corneal degeneration, glaucoma or uveitis. We excluded keratoconic eyes from this study by using the keratoconus screening test equipped with the corneal topographer (TMS-4, Tomey, Nagoya, Japan). This retrospective clinical chart review was approved by the Institutional Review Board at Kitasato University Hospital (B20-101), and followed the tenets of the Declaration of Helsinki. Our Institutional Review Board waived the requirement for informed consent for this review of the charts.

### Surgical procedure

ICL exchange (extraction followed by implantation of another ICL lens) was performed as follows: On the day of surgery, dilating and topically anesthetic agents were administrated. After a new 3.0-mm temporal corneal incision was made, we filled the anterior chamber with a viscoelastic substance, and then the proximal ICL haptics were displaced, grasped with forceps, and removed through the incision. The replacement ICL (different power or different size) was inserted into the posterior chamber, the viscoelastic substance was replaced with a balanced salt solution, and a miotic agent was administrated.

ICL extraction and subsequent cataract surgery were performed as follows: After ICL extraction as mentioned above, standard phacoemulsification consisted of capsulorrhexis, nuclear and cortex extraction, and monofocal IOL implantation using the same incision. The IOL power was calculated by using the Barrett Universal II formula without any adjustment. We topically used steroidal (0.1% betamethasone) and antibiotic (1.5% levofloxacin) medications 4 times daily for 1 week, the dose being reduced gradually thereafter.

### Assessment of outcomes and patient satisfaction

We calculated the percentage and the etiology of the patients who required ICL extraction among all current ICL-implanted patients. The type and the location of cataract formation were determined by a slit-lamp examination after mydriasis. Preoperatively and 3 months postoperatively, we assessed the logarithm of minimal angle of resolution (logMAR) UDVA, CDVA, the percentages of eyes within ± 0.5 and 1.0 D of the targeted correction, ECD, and patient satisfaction, in such ICL-extracted eyes. The ECD was measured using a non-contact specular microscope (SP-8800, Konan, Nishinomiya, Japan). Patient satisfaction was determined using a visual analogue scale, in a range from 0 (very dissatisfied) to 10 (very satisfied), by one examiner who did not participate in the overall treatment or follow-up of the patients in this study.

### Statistical analysis

Since we confirmed that the data were not normally distributed by the Shapiro–Wilk test, the Wilcoxon signed-rank test was utilized to compare the pre- and post-surgical data. The results are described as mean ± standard deviation, a value of p < 0.05 was deemed statistically significant.
